# Predictors of long-term post-thrombotic syndrome following high proximal deep vein thrombosis: a cross-sectional study

**DOI:** 10.1186/s12959-020-00253-8

**Published:** 2021-01-08

**Authors:** Marit Engeseth, Tone Enden, Per Morten Sandset, Hilde Skuterud Wik

**Affiliations:** 1 Institute of Clinical Medicine, University of Oslo, Oslo, Norway; 2 Department of Haematology, Oslo University Hospital, P.O.Box 4950 Nydalen, N-0424 Oslo, Norway; 3 Division of Radiology and Nuclear medicine, Oslo University Hospital, Oslo, Norway

**Keywords:** Comorbidity, Deep vein thrombosis, Post-thrombotic syndrome, Predictors, Venous insufficiency

## Abstract

**Background:**

Post-thrombotic syndrome (PTS) is a frequent chronic complication of proximal deep vein thrombosis (DVT) of the lower limb, but predictors of PTS are not well established. We aimed to examine predictors of PTS in patients with long-term PTS following proximal DVT.

**Methods:**

During 2006–09, 209 patients with a first time acute upper femoral or iliofemoral DVT were randomized to receive either additional catheter-directed thrombolysis or conventional therapy alone. In 2017, the 170 still-living participants were invited to participate in a cross-sectional follow-up study. In the absence of a gold standard diagnostic test, PTS was defined in line with clinical practice by four mandatory, predefined clinical criteria: 1. An objectively verified DVT; 2. Chronic complaints (> 1 month) in the DVT leg; 3. Complaints appeared after the DVT; and 4. An alternative diagnosis was unlikely. Possible predictors of PTS were identified with multivariate logistic regression.

**Results:**

Eighty-eight patients (52%) were included 8–10 years following the index DVT, and 44 patients (50%) were diagnosed with PTS by the predefined clinical criteria. Younger age and higher baseline Villalta score were found to be independent predictors of PTS, i.e., OR 0.96 (95% CI, 0.93–0.99), and 1.23 (95% CI, 1.02–1.49), respectively. Lack of iliofemoral patency at six months follow-up was significant in the bivariate analysis, but did not prove to be significant after the multivariate adjustments.

**Conclusions:**

In long-term follow up after high proximal DVT, younger age and higher Villalta score at DVT diagnosis were independent predictors of PTS.

## Background

Post-thrombotic syndrome (PTS) is a long-term complication in up to 50% of patients with a previous proximal deep vein thrombosis (DVT) [1–3]. PTS is a chronic syndrome with various intermittent or persistent symptoms and signs of the lower limb; typically skin discoloration, swelling, and heaviness, and in severe cases painful venous ulcers and activity limiting venous claudication [4–8]. PTS is associated with a reduction in quality of life (QoL), high demands on health care costs, and there are few treatment options [9–12].

There is no gold standard objective test for diagnosing PTS [13]. In clinical practice, patients who present with typical lower limb symptoms and signs later than 3–6 months following an acute DVT, and with no other obvious cause, are considered to have developed PTS [14, 15]. On the other hand, in clinical trials various scorings systems have been used for the diagnosing, grading, and follow-up of PTS [13, 16–18]. This is problematic when comparing results across studies and when implementing research for improved post-thrombotic care [19, 20]. In 2009, the International Society on Thrombosis and Hemostasis (ISTH) recommended the Villalta scale [13, 17] for PTS assessment in clinical research. In the absence of a gold standard test, the Villalta scale has been validated through correlations to generic and disease-specific QoL scores [10, 21–23], and anatomic and physiologic markers [20, 24, 25].

Despite being the recommended scoring system, limitations in the diagnostic accuracy of the Villalta scale have been recognized [26–29]. The scale is unspecific and includes symptoms and signs that may occur in various other diseases [26]. Moreover, some patients with severe leg problems that obviously relate to the DVT, i.e., they appeared in the same leg with no other obvious cause(s), may not qualify as PTS by the Villalta score [13, 28, 30, 31]. This typically applies to patients with activity limitations due to venous claudication [18, 27]. In a recent qualitative study, we explored the relations between the different items of the Villalta scale and the clinical characteristics and complaints described by patients with moderate and severe PTS. Our findings indicated that the Villalta scale does not capture various typical PTS complaints or reflect on the importance of PTS symptoms and signs [5]. Thus, an improved diagnostic approach for PTS seems warranted. In the absence of a gold standard diagnostic test, we used four mandatory predefined clinical PTS criteria in line with how we diagnose PTS in clinical practice [5, 32, 33]. We included any chronic complaint that had developed in the index limb following the objectively verified acute DVT, and an alternative diagnosis for the complaints had to be unlikely; hereunder excluding any preexisting comorbidity of the leg (Table 1).

**Table 1:** Four mandatory and predefined clinical criteria for PTS

1. Previous objectively verified DVT 2. Development of chronic complaints (> 1 month) in the DVT leg*	
3. The chronic complaints appeared or worsened following the DVT	
4. An alternative diagnosis to the patient’s complaints is not likely	

*Any chronic complaint developed in the limb following the DVT [5, 32]

As there is no curative treatment for PTS [17], identification of predictors of PTS may help identifying patients at risk of developing PTS, facilitate PTS prevention, and guide in the development toward improved PTS management and care [34]. Previous studies of PTS predictors have mainly used the Villalta scale for PTS assessment [1, 2, 29, 35–41]. However, the predictors identified have not been consistent across studies and have included proximal DVT, recurrent ipsilateral DVT, provoked DVT, pre-existing chronic venous insufficiency, obesity, smoking, older age, thrombophilia, deep venous reflux, and persistent venous obstruction [17, 34, 42]. Some studies have found men to be at higher risk; others have found the opposite [2, 43, 44]. Recurrent ipsilateral DVT is a strong predictor in a number of these studies, however, the magnitude of the effect differs across the studies, which can be explained by variability in study populations, and PTS definitions [17].

In the current study, we aimed to examine predictors of long-term PTS as defined by the four predefined clinical criteria in line with clinical practice [5, 32].

## Methods

### Study population

During 2006–2009, the Catheter-directed Venous Thrombolysis (CaVenT) study randomized 209 participants with a first-time upper femoral and/or iliofemoral DVT to receive either additional catheter-directed thrombolysis (CDT) or conventional anticoagulant treatment. The protocol, and main outcomes from two and five years follow-up have been reported elsewhere [3, 42]. In 2017, we re-invited the 170 CaVenT participants who were still alive to participate in a cross-sectional follow-up study of long-term complications. A detailed description of the population has previously been published [3, 42].

### Variables and instruments

At the study visit PTS was assessed with the four predefined criteria (Table 1) by an experienced study investigator (ME).

Various baseline variables from the CaVenT study were included, hereunder age, gender, smoking, and the duration of symptoms before DVT diagnosis. Localization of baseline DVT based on routine diagnostic imaging was categorized as isolated pelvic, iliofemoral, or upper femoral DVT. For predictor analysis, a variable “DVT with pelvic involvement” included both iliofemoral and isolated pelvic DVT. Other baseline variables included allocated treatment arm within the CaVenT study, D-dimer, C-reactive protein (CRP), Villalta score, and increased calf circumference. For predictor analysis, we registered D-dimer ≥0.5 μg/mL and CRP ≥ 4 mg/L, i.e., above the reference level. Differences in calf circumference were measured in centimeters at the level of the tuberosity of the tibia between the index leg and the contralateral leg. At baseline, DVT was categorized as provoked if occurring following trauma, immobilization, or surgery, and in patients with cancer, hormone-replacement therapy (HRT), or recent pregnancy. Variables from the six months follow-up in the CaVenT study used as possible predictors in the present study included femoropopliteal reflux, i.e., venous incompetence with reflux defined as venous valve closure time > 0.5 m/sec on duplex ultrasound, and iliofemoral patency, i.e., flow in the pelvic and femoral vein with complete compressibility of the femoral vein and no functional venous obstructions, assessed by ultrasound and air plethysmography [40]. Finally, we included daily use of elastic compression stockings (ECS), and recurrent ipsilateral DVT from two and five years follow up.

### Statistical analyses

Continuous variables (i.e., age, differences in leg circumference, and duration of symptoms before DVT diagnosis) are presented as mean with standard deviation (SD). Dichotomous variables (i.e., gender, daily smoking, DVT with pelvic vein involvement, left sided DVT, additional CDT, and daily use of ECS) are presented as frequencies with percentages (Table 2). To identify possible predictors of PTS, we tested whether known risk factors for PTS, additional CDT treatment, differences in leg circumference, lack of iliofemoral patency, and presence of iliofemoral reflux were differently distributed between patients with and without PTS using bivariate logistic regression. Continuous variables (i.e., age, Villalta score at baseline, duration of symptoms before DVT diagnosis, and difference in leg circumference) were not dichotomized in these analyses, but kept as continuous variables. The Wald test was used to test the level of significance. All variables with *P*-values < 0.20 in the bivariate analyses were included in a multivariate logistic regression model with backward variable selection, until all the remaining variables were statistically significant. Differences with a P-value < 0.05 were considered statistically significant. Results from the bivariate and multivariate logistic regression were presented as odds ratios (OR) with 95% confidence intervals (CI). Variables that were significant in the final model were also checked for interactions. Statistical Package for Social Sciences (SPSS) version 25 was used for all analyses (SPSS Inc., Chicago, Illinois, USA).

**Table 2:** Demographic and clinical characteristics

*Baseline*	*N* = 88
Age, years	50.5 (15.5)
Women	31 (35)
Daily smoking	18 (20.5)
Duration of symptoms before DVT diagnosis, days	6.8 (5.0)
Localization of DVT*	
Isolated pelvic	3 (3.4)
Iliofemoral	40 (45.5)
Upper femoral	42 (47.7)
Left-sided DVT	49 (55.7)
Allocated treatment group in the CaVenT study	
Conventional anticoagulant treatment	45 (51)
Additional catheter-directed thrombolysis	43 (49)
*Follow-up data, CaVenT*	
Daily use of elastic compression stockings	68 (77.3)
Recurrent ipsilateral DVT†	10 (11.4)

Data are from the randomized clinical trial; the Catheter-directed Venous Thrombolysis (CaVenT) study.

Continous data are presented as mean (SD) and categoric data as number (%).

*Based on routine diagnostic imaging at baseline, *n* = 85

†Data from 24 to 60 months follow-up.

## Results

### Demographic and clinical characteristics

Eighty-eight (52%) of the eligible CaVenT study participants were included during October 2017–June 2018. Thirty-one (35%) of the participants were female. Mean age was 60.7 (SD 15.4) years, and mean follow-up time since index DVT was 9.5 (SD 1.2) years. Localization of index DVT, as well as other baseline and follow-up characteristics, are presented in Table 2. Based on data from the CaVenT study, the characteristics of the 88 participants and the 82 non-participants were comparable (i.e., age at DVT diagnosis, gender, localization of index DVT, PTS diagnosed by the Villalta scale at 5 years follow up, and treatment group in the CaVenT study except for comorbidity of the index leg which was present in 32 (36%) of non-participants compared to 11 (12.5%) of participants (*p* < 0.001). Other details have been presented elsewhere [3, 42].

When assessing PTS by the mandatory predefined clinical criteria, PTS was diagnosed in 44 (50%) of the participants. Chronic complaints in the DVT limb included activity-related discomfort, pressure, aching, impaired sensitivity/paresthesia, venous ectasia, hyperpigmentation, redness, venous ulcers, heaviness, and edema. We excluded preexisting complaints including sequela after fractures, sequelae from trauma and stroke, arthrosis, varicose veins, and psoriasis arthritis. There was statistically no significant difference of PTS diagnosed by the predefined clinical criteria in the participants receiving additional CDT versus conventional anticoagulant treatment, i.e., 19 (44%) versus 25 (56%), respectively (*p* = 0.3).

### Predictors of post-thrombotic syndrome

Younger age, higher baseline Villalta score, and lack of iliofemoral patency at six months follow-up were significantly associated with PTS in the bivariate analyses. Participants with recurrent ipsilateral DVT, and smokers, respectively, were too few to be assessed. Both DVT with pelvic vein involvement and provoked DVT had *p*-values < 0.20 in the bivariate analyses and were included in the multivariate model. Gender, treatment with additional CDT, D-dimer > 0.5 μg/mL, CRP ≥ 0.4 mg/L, increased leg circumference at DVT diagnosis, and duration of symptoms before DVT diagnosis, were not associated with PTS in the bivariate analyses (Table 3). In the multivariate model, the two continuous variables younger age and higher baseline Villalta score were independent predictors of PTS. Age was negatively associated with PTS with an OR of 0.96 (95% CI, 0.93–0.99), hence younger age was associated with the development of PTS. A higher symptom burden at the time of the DVT diagnosis, as assessed by the Villalta score, was associated with PTS with an OR of 1.23 (95% CI, 1.02–1.49). We found no interactions between these two continuous variables.

**Table 3:** Crude and adjusted odds ratios for post-thrombotic syndrome

Variable	*N*	Crude OR (95% CI)	*P*- value*	Adjusted OR (95% CI)	Adjusted *P*-value*
Baseline					
Age (continuous), years	88	0.96 (0.94–0.99)	**0.01**	0.96 (0.93–0.99)	**0.01**
Women	88	0.74 (0.30–1.78)	0.50		
Daily smoking	88	1.32 (0.46–3.74)	0.59		
Duration of symptoms before DVT diagnosis, days	88	1.02 (0.94–1.11)	0.55		
DVT with pelvic vein involvement	85	2.09 (0.87–5.03)	**0.10**		
Left-sided DVT	88	1.45 (0.62–3.38)	0.39		
Additional catheter-directed thrombolysis	88	1.57 (0.68–3.66)	0.28		
Provoked DVT	88	1.94 (0.76–4.95)	**0.16**		
Villalta score (continuous)	83	1.23 (1.02–1.41)	**0.03**	1.23 (1.02–1.49)	**0.03**
Differences in leg circumference (continuous), cm	65	0.89 (0.71–1.13)	0.36		
D-dimer ≥0.5 μg/mL	83	0.65 (0.10–4.09)	0.65		
CRP ≥4 mg/L	82	0.46 (0.10–1.98)	0.30		
Follow-up data, CaVenT					
Daily use of ECS	76	1.48 (0.32–6.69)	0.61		
Lack of iliofemoral patency	86	2.88 (1.18–7.01)	**0.02**		
Femoropoplietal reflux	87	1.14 (0.47–2.77)	0.76		

*DVT* deep vein thrombosis, *ECS* elastic compression stockings, *OR* odds ratio, *CI* confidence interval, Wald test*.

## Discussion

When defining PTS by four mandatory and predefined clinical criteria in line with clinical practice, we found that younger age and a higher baseline Villalta score were independent predictors of long-term PTS in patients with a prior high proximal DVT.

Our findings are likely to contribute to the limited knowledge of predictors for long-term PTS, and identifying patients at risk is an important step towards developing improved treatment strategies for the prevention of PTS [17, 45]. Also, our findings may indicate that PTS remains a common chronic complication for up to ten years following a first-time high proximal DVT. Younger age was an independent predictor of PTS which is in contrast to previous studies reporting that older age is a predictor for PTS [1, 2, 44, 46, 47]. Lower leg comorbidity is likely to be more common in older patients, and may have contributed to confounding in previous studies. This possible confounding was avoided in our study as obvious comorbid leg symptoms and signs excluded PTS according to the predefined criteria.

The baseline Villalta score is likely to indicate the symptom burden at the time of DVT diagnosis, and a higher baseline score was an independent predictor of long-term PTS. Others have also found that a higher baseline Villalta score predicts a less favorable long-term outcome. Rabinovich et al. developed a prediction model for PTS based on the SOX study and found that patients with a Villalta score ≥ 4 at baseline, in addition to pelvic DVT and body mass index ≥35 kg/m^2^, predicted increased risk of PTS [48]. In addition, Kahn et al. found that higher Villalta score at one-month post-DVT was a strong predictor for PTS [2].

Previous studies have also found iliofemoral DVT [2, 44], recurrent ipsilateral DVT [2, 39, 46, 49], and high body mass index (BMI) [29] as strong predictors for PTS [44, 48]. DVT with pelvic vein involvement were included in our multivariate model but was not a significant predictor in our study. The incidence of recurrent ipsilateral DVT was low, and data on baseline BMI was collected only in a subset of patients. Thus, neither of these two variables could be evaluated in the present study.

The introduction of CDT for PTS prevention was based upon the “open vein hypothesis”, where an accelerated thrombus removal is thought to prevent venous valve incompetence and incomplete recanalization [3, 50]. At the six months follow-up of the CaVenT trial [42] the lack of iliofemoral patency and deep venous reflux were found to be independent predictors of PTS in the treatment group receiving CDT; and PTS was defined by the Villalta scale [40]. Prandoni et al. reported an increased risk of PTS in patients with persistent venous obstruction or popliteal reflux at six months follow-up [25]. In the current study, neither lack of iliofemoral patency nor deep venous reflux came out as independent predictors of PTS. This could be due to selection bias as only 52% of the still eligible patients were included, the lack of power, or the use of a different definition of PTS.

It is acknowledged that the clinical presentation of PTS is non-specific and conditions as primary venous insuffiency, trauma, central venous hypertension, and arthrosis may present with similar clinical manifestations [26, 32]. Moreover, reported predictors of PTS are overlapping with predictors of primary venous insufficiency [30, 34]. Galanaud et al. used data from the REVERSE study to assess risk factors for PTS in patients with a first unprovoked proximal DVT who were free of clinically significant primary venous insufficiency with an effort to remove biased evaluation of PTS. After excluding patients with primary venous insufficiency, only obesity remained an independent predictor of PTS [29]. Previous studies have shown that Villalta scores in the ipsilateral and contralateral legs are strongly correlated, indicating that cases considered as PTS may reflect pre-existing chronic venous disease [17, 26, 51, 52]. When assessing PTS by the four clinical criteria, we excluded patients with complaints likely to be explained by primary venous insufficiency and other lower limb comorbidity.

A major limitation of this study includes the PTS assessment with no previously validated diagnostic tool. We assessed PTS in line with clinical practice [5, 26, 27, 32]. The reported chronic complaints in the present study were to a great extent included in the Villalta scale. However, the scale does not include the typical pain and tightness in the thigh/calf during exercise with relief during rest, i.e., venous claudication which is often seen in persistent vein obstruction following iliofemoral DVT [53]. Furthermore, the Villalta scale does not require symptoms or signs to be chronic, or consider whether the complaints could be explained by other conditions, i.e., comorbidity, we consider the four mandatory predefined clinical criteria to be relevant and contribute to improved diagnostic accuracy. However, without a previously validated diagnostic tool, our findings can only be considered as preliminary, hypothesis generating, and further research is needed.

Another limitation was that one study investigator performed all study visits and assessments. As only 52% of the eligible patients gave consent to participate in the current study there is a possibility for selection bias, however the participants and non-participants did not differ except for more leg comorbidity among non-participants [32]. Comparisons between DVT with and without pelvic involvement in our material are uncertain, as pelvic involvement was not routinely assessed in patients randomized to the control group [3]. Moreover, we were not able to assess BMI as patients’ height and weight were not systematically collected at baseline. We do not have data on time in therapeutic range (TTR), thus, we were not able to evaluate the quality of anticoagulation as a possible predictor for PTS. A strength of the current study is the long follow-up time.

## Conclusions

We conclude that younger age and higher Villalta score at the time of DVT diagnosis are independent predictors of long-term PTS.

## Data Availability

The datasets used in the current study are available from the corresponding author on reasonable request.

## Abbreviations

BMI: Body mass index
CaVenT: Catheter-directed Venous Trombolysis
CDT: Catheter-directed thrombolysis
CI: Confidence interval
CRP: C-reactive protein
DVT: Deep vein thrombosis
ECS: Elastic compression stockings
ISTH: International Society on Thrombosis and Haemostasis
HRT: Hormone-replacement therapy
OR: Odds ratio
QoL: Quality of life
SPSS: Statistical Package for Social Sciences
PTS: Post-thrombotic syndrome
